# DEIA is essential to advance the goals of translational science: Perspectives from NCATS

**DOI:** 10.1017/cts.2022.482

**Published:** 2022-11-09

**Authors:** Shadab F. Hussain, Amanda L. Vogel, Jessica M. Faupel-Badger, Linda Ho, Lameese D. Akacem, Krishna Balakrishnan, Rebekah Geiger, Rashmi Gopal-Srivastava, Brittany Haynes, Marcus G. Hodges, Tanetta Isler, Ewy A. Mathé, Leonie Misquitta, Karlie R. Sharma, Eric Sid, Jamie L. Zigterman, Penny W. Burgoon

**Affiliations:** National Center for Advancing Translational Sciences, Bethesda, MD, USA

**Keywords:** Translational science, diversity, equity, inclusion, accessibility, health disparities, workforce diversity, community engagement

## Abstract

The National Center for Advancing Translational Science (NCATS) seeks to improve upon the translational process to advance research and treatment across all diseases and conditions and bring these interventions to all who need them. Addressing the racial/ethnic health disparities and health inequities that persist in screening, diagnosis, treatment, and health outcomes (e.g., morbidity, mortality) is central to NCATS’ mission to deliver more interventions to all people more quickly. Working toward this goal will require enhancing diversity, equity, inclusion, and accessibility (DEIA) in the translational workforce and in research conducted across the translational continuum, to support health equity. This paper discusses how aspects of DEIA are integral to the mission of translational science (TS). It describes recent NIH and NCATS efforts to advance DEIA in the TS workforce and in the research we support. Additionally, NCATS is developing approaches to apply a lens of DEIA in its activities and research – with relevance to the activities of the TS community – and will elucidate these approaches through related examples of NCATS-led, partnered, and supported activities, working toward the Center’s goal of bringing more treatments to all people more quickly.

## Introduction

In December 2021, the National Center for Advancing Translational Sciences (NCATS) celebrated its 10^th^ anniversary with a virtual commemoration event. At this event, NCATS Director, Dr. Joni Rutter, introduced aspirational goals over the next decade for NCATS to change from bringing “more treatments to more people more quickly” to bringing “more treatments to *all* people more quickly.” In her first NCATS Director’s message of 2022, Dr. Rutter affirmed the Center’s commitment to “tackle health disparities in all areas we support, while also building an organizational culture that supports diversity, equity, inclusion, and accessibility” [1]. This commitment is aligned with broader efforts in the translational science community [2] and across the National Institutes of Health (NIH) to confront and address health inequities and structural racism in biomedical research [3, 4].

To ensure equitable access for all communities and the advances translational science has to offer, maximum incorporation of diversity, equity, inclusion, and accessibility (DEIA) must be a central concern for translational science (see Table 1 for definitions). Translational science is the field of investigation focused on understanding scientific and operational principles underlying the full translational process [5]. It seeks to identify and address bottlenecks that are common across the translational spectrum and identify solutions that increase the efficiency and impact of translational research. In working toward combating health inequities, we must ask ourselves a range of questions when examining the translational process through a DEIA lens:

**Table 1.** Definitions of terms utilized in NIH workforce diversity and health disparities-related activities

Term	Definition
Diversity^a^	The practice of including the many communities, identities, races, ethnicities, backgrounds, abilities, cultures, and beliefs of the American people, including underserved communities.
Equity^a,b^	The consistent and systematic fair, just, and impartial treatment of all individuals, including individuals who belong to underserved communities that have been denied such treatment, such as Black, Latino, and Indigenous and Native American persons, Asian Americans and Pacific Islanders, and other persons of color; members of religious minorities; lesbian, gay, bisexual, transgender, and queer (LGBTQ+) persons; persons with disabilities; persons who live in rural areas; and persons otherwise adversely affected by persistent poverty or inequality.
Inclusion^a^	The recognition, appreciation, and use of the talents and skills of employees of all backgrounds.
Accessibility^a^	The design, construction, development, and maintenance of facilities, information and communication technology, programs, and services so that all people, including people with disabilities, can fully and independently use them.
Health disparities^c^	A health disparity is a health difference that adversely affects disadvantaged populations, based on one or more of the following health outcomes: • Higher incidence and/or prevalence and earlier onset of disease • Higher prevalence of risk factors, unhealthy behaviors, or clinical measures in the causal pathway of a disease outcome • Higher rates of condition-specific symptoms, reduced global daily functioning, or self-reported health-related quality of life using standardized measures • Premature and/or excessive mortality from diseases where population rates differ • Greater global burden of disease using a standardized metric
Minority health^c^	Refers to the distinctive health characteristics and attributes of racial and/or ethnic minority groups, as defined by the U.S. Office of Management and Budget, that can be socially disadvantaged due in part to being subject to potential discriminatory acts.
Underrepresented populations^d^	As defined by the U.S. Biomedical, Clinical, Behavioral, and Social Sciences Research Enterprise: (1) Individuals from racial and ethnic groups that have been shown by the National Science Foundation to be underrepresented in health-related sciences on a national basis; (2) Individuals with disabilities, who are defined as those with a physical or mental impairment that substantially limits one or more major life activities, as described in the Americans with Disabilities Act of 1990, as amended; and (3) Individuals from disadvantaged backgrounds, defined as those who meet two or more of the criteria listed in the Notice.

a Executive order 14,035 on diversity, equity, inclusion, and accessibility in the federal workforce.

b Executive order 13,985 on advancing racial equity and support for underserved communities through the federal government.

c NIH minority health disparities strategic plan.

d NIH’s notice of interest in diversity (NOT-OD-20-031).

- How do we ensure critical decision-making in the scientific process (e.g., asking the right questions, identifying solutions, being accountable to our organization) includes diverse perspectives?
- How do we ensure research outcomes have relevance to the full diversity of the US population?
- How do we engage diverse populations and communities to increase involvement in research, both as collaborators and participants?
- How do we ensure equitable access to translational solutions (e.g., treatments, cures, interventions)?

Viewing translational science through a DEIA lens can illuminate translational roadblocks across all stages of research, among all diseases and conditions, and ensure relevance of solutions to the community. It is imperative that workforce diversity is prioritized along with the research process. Past research shows that aspects of DEIA, particularly diverse research teams, can positively impact creativity and innovation in science [6], and ultimately scientific quality [7, 8]. Furthermore, diverse research teams and scientists from underrepresented backgrounds are more likely to focus on research topics at the community and population level and may be more capable of addressing health disparities given this community-level research focus and exploring social determinants of health [9, 10]. Elevating DEIA as a core goal of translational science has the potential to transform how translational research is conducted and how outcomes are relevant to the diversity of the US population.

In this paper, we describe ongoing efforts toward applying a DEIA lens across NCATS workforce and research activities. We also highlight core issues for consideration and share examples of how these considerations are being assessed in NCATS programs, operations, and practices. This paper outlines ongoing and planned efforts that illustrate NCATS’ interest and commitment to efforts that support DEIA with an understanding that these efforts will also support the Center’s mission to bring more treatments to *all* people more quickly.

### NCATS Workforce Diversity

In Summer 2020, aligned with global protests against anti-Black racism, self-assembled employee groups of Black NIH staff, who were motivated to ensure the NIH workplace culture is one free of racism, discrimination, and harassment, penned an anonymous open letter to NIH Director Francis Collins, called the Eight Changes for Racial Equity (8CRE), to address racial inequities within the NIH workforce [3]. The letter highlighted how structural racism impacted the NIH’s overall mission to enhance health and shared eight strategies to improve racial equity within the NIH. Dr. Collins met with 8CRE supporters to discuss their strategies. In response to 8CRE and other issues of racism in the workplace and structural disadvantages reported by those typically underrepresented in the biomedical research workforce, Dr. Collins formed the UNITE initiative in February 2021 [4]. The UNITE initiative is a commitment by all 27 NIH Institutes and Centers and the Office of the Director to end structural racism in biomedical research. It consists of five subcommittees, each tasked with a unique focus area (see Table 2), engaging over 90 staff members across NIH, including representatives from NCATS.

**Table 2.** List of NIH and NCATS internal efforts addressing workforce diversity and health disparities

Activity	Mission/vision	Major results to-date
NIH-wide activities		
8CRE (8 Changes for Racial Equity) Letter^a^ Created in July 2020	Mission: To promote a culture where Black and other racially and ethnically underrepresented employees are respected, valued, treated fairly and with equity. Vision: A workplace free of racism and discrimination at NIH that embraces diversity, equity, and inclusivity.	Open letter to NIH Director addressing 8 changes to improve racial equity in the workplace Spurred formation of NIH-wide and Institute/Center committees (e.g., UNITE initiative) addressing workforce diversity and health disparities research
UNITE Initiative^b^ Formed in February 2021	NIH-wide initiative consisting of subcommittees focused on: U – Understanding stakeholder experiences through listening and learning N – New research on health disparities, minority health, and health equity I – Improving the NIH culture and structure for equity, inclusion, and excellence T – Transparency, communication, and accountability with our internal and external stakeholders E – Extramural research ecosystem: changing policy, culture, and structure to promote workforce diversity	Published a variety of funding opportunities throughout the agency related to health disparities and health inequities. Modified internal workforce policies including those related to individual performance plans to include a component in advancing DEIA. Expanded programs such as the Science and Education Partnership Award program to focus on expanding career opportunities for those from underrepresented populations. See more accomplishments on UNITE Initiative website.
NCATS activities		
NCATS Inclusion, Diversity, and Equity in Action (IDEA) Council Formed in July 2020	Mission: Cultivate a deep culture of belonging for all people at NCATS by celebrating diversity, ensuring equity, and promoting inclusion of all voices to fulfill the NCATS mission. Vision: Create a culture of inclusive excellence through holistic and innovative approaches to incorporating diversity, equity, and inclusion into all NCATS activities.	Held listening sessions with NCATS staff focused on DEIA-related issues Analyzed and internally shared Federal Employee Viewpoint Survey workforce climate data Recommended training in systemic racism for all supervisors
NCATS Health Disparities Working Group (HDWG) Formed in July 2020	Mission: Provide NCATS with recommendations for using translational science to eliminate health disparities by: • Assessing current center activities, • Identifying opportunities to advance health equity, • Providing guidance on best practices and resources, and • Setting benchmarks to ensure that NCATS continues to be accountable toward these goals. Vision: As a tenet of NCATS culture, health equity drives all NCATS program/project development and priority setting activities to leverage translational science that advances medical outcomes for marginalized populations	Conducted all-staff survey 25% response rate and follow-up interviews with 5% of the workforce selected for broad representation of the diversity of NCATS divisions, offices, roles, and demographic characteristics. Based on the survey and interview results, produced a report offering a landscape analysis of current and planned NCATS activities that either: (a) currently have implications for advancing work on minority health, health disparities, or health equity (MH/HD/HE); or (b) could be leveraged, expanded or modified to have implications for MH/HD/HE. Developed an action plan for advancing NCATS investments in DEIA across our activities, including measurable goals and objectives.

a 8cre website: https://www.support8cre.com/.

b UNITE website: https://www.nih.gov/ending-structural-racism/unite.

In alignment with NIH, NCATS is examining its internal and external activities to identify and address practices that can perpetuate systemic bias and structural racism in the biomedical research workforce. This examination includes analyzing workforce diversity in both internal and external areas and identifying approaches to create inclusive environments for all members of the workforce. NCATS is working toward advancing DEIA in the translational science workforce through three activities: (1) forming the internal NCATS Inclusion, Diversity, and Equity in Action (IDEA) Council to examine NCATS’ internal workforce diversity and culture; (2) creating education and training initiatives; and (3) disseminating extramural research grants.

### NCATS Inclusion, Diversity, and Equity in Action (IDEA) Council and Organizational Culture Change

Formed in July 2020, the NCATS IDEA Council focuses on the internal workforce and culture at NCATS. Members represent all NCATS Offices and Divisions, include both scientific and administrative positions, and are diverse by race/ethnicity and gender identity. The vision of this Council is to create a culture of belonging for people at NCATS and of inclusive excellence through holistic and innovative approaches to incorporating DEIA into NCATS activities.

The IDEA Council has quickly turned its efforts toward assessing the results of NCATS employee responses to the Federal Employee Viewpoint Survey (FEVS), an annual climate survey of the Federal workforce. The FEVS shares high-level results, including employee gender and racial demographic categories. NCATS reviewed the 2020 FEVS data, identifying baseline findings by demographic group, and the Council reported these findings to NCATS leadership and to staff through all-hands town hall meetings and reports accessible to all staff. Results have been used by NCATS administration to identify specific areas for intervention (e.g., workload balance) and areas for continued improvement (e.g., recognizing high-quality performance). Due to the COVID-19 pandemic, the FEVS was conducted with different questions in 2021 and NCATS-level data were not available. NCATS is awaiting the 2022 data and plans to review that data by gender and racial demographic categories. NCATS will be able to compare the 2022 data to the 2020 baseline to examine any change in results.

Additionally, the NIH is aligning its efforts with President Biden’s Executive Order 13,985 to enhance racial equity through Federal Government policies [11]. Thus, NCATS, in concert with NIH-wide activities, developed an internal plan with three focus areas: (1) Anti-Racism Education and Engagement; (2) Integrating DEIA into the NCATS Strategic Plan; and (3) Recruitment and Retention of a Diverse Workforce. The IDEA Council has planned NCATS-wide events and trainings on DEIA. Dr. Marie Bernard, the NIH Chief Officer for Scientific Workforce Diversity, was invited to present her vision and discuss evidence-based practices for enhancing scientific workforce diversity. The talk was widely attended and NCATS staff participated in facilitated discussion groups focused on how NCATS could use Dr. Bernard’s vision to guide NCATS activities.

Subsequently, the IDEA Council organized a two-day, NCATS Supervisory staff training focused on historical and institutional racism in the United States. The goal was to establish a shared understanding of the foundations of structural racism in the United States to support discussions regarding approaches to advance DEIA across NCATS activities. Results from a post-evaluation survey of 43 out of 69 attendees revealed that, although the training was perceived as being dense with information by 35% of respondents, 67% enjoyed learning about the historical context of systemic racism and 85% were eager to learn more and address systemic racism in the workplace. Additionally, the training was successful in increasing the number of attendees from 33% pre-training to 62% post-training who indicated they had knowledge of systemic racism and how it relates to NCATS. Lastly, 74% of attendees were eager to attend more DEIA trainings. The overall positive response to this training with supervisors led to further targeted anti-racism training for supervisors and plans to offer similar trainings to all NCATS staff. NCATS staff has also participated in an NIH-wide training focused on addressing microaggressions in the workplace which was received positively by attendees, and this training was held again in Fall 2022 specifically for NCATS staff.

Currently, the Council is building on these efforts through establishing workgroups to continue analyzing workforce data, plan staff educational activities, create and implement plans to diversify the translational science workforce, and develop goals for future activities. Additionally, the Council is working with an outside contractor to identify areas of strength and improvement within NCATS in building the Center’s plans to incorporate DEIA in its practices. Altogether, these efforts aim to integrate DEIA into the Center culture and empower staff to proactively incorporate DEIA into their roles. All initiatives are rigorously evaluated by Center staff to ensure DEIA initiatives are successful and continuously improved.

### Education and Training Opportunities

Activities to diversify the translational science workforce will also benefit from expanded education and training opportunities that offer a broader range of entry points. This will build upon existing NCATS education and training resources that have been focused on individuals at the doctoral, postdoctoral, and early-career faculty levels [12, 13]. Expanding these efforts, the NCATS Education Branch has recently developed opportunities accessible to many education and career stages. For example, the branch currently offers online courses designed for an audience with broad scientific knowledge, to bring awareness of translational science to interested individuals outside traditional academic settings [14].

Additionally, in Spring 2022, NCATS joined the NIH Science Education Partnership Awards (SEPA) Funding Opportunity Announcement supporting science education activities for students ranging from pre-kindergarten to grade 12. The overall goals of this program are to encourage individuals from diverse backgrounds, including those from underrepresented groups, to pursue further studies or careers in biomedical, behavioral, and clinical research, and help these individuals build their understanding of research and its implications. In its over 30-year history, SEPA has successfully enhanced interest in future science education and careers among diverse students [15]. NCATS’ participation adds translational science as a new area of emphasis for this program.

In addition, NCATS offers undergraduate, postbaccalaureate, graduate, and postdoctoral research training opportunities on the NIH campus at the NCATS intramural research laboratories [16]. An eight-week summer internship program for undergraduates offers hands-on experiential research training, professional development, career exploration, and a translational science course. Beginning in 2022, the summer internship program will include the NCATS GREATS (Gaining Research Equity and Advancement in Translational Sciences) Program, which leverages a cohort model to support a diverse group of undergraduate and graduate student participants. In addition to the core internship experience, GREATS provides seminars designed to develop skills in translational science, and opportunities to network with NCATS researchers to inform training and career pathways. In Summer 2022, two interns participated in the GREATS program in a cohort of 15 summer interns. Evaluation of the program is ongoing, and the GREATS program will be offered again in Summer 2023.

### Extramural Workforce

NCATS provides funding to support extramural clinical and translational research in the Clinical and Translational Science Awards (CTSA) Program, which provided over $586 million in support to 61 research institutions in fiscal year 2021 [17]. The CTSA Program provides funding to support recruitment and retention at multiple points in the biomedical research career path. Notably, the program is committed to enhancing the diversity of affiliated trainees and investigators through the Diversity, Re-entry, and Reintegration Research Administrative Supplements [18]. More information describing the racial/ethnic demographic composition of CTSA investigators can be found in a separate article in this special issue [19].

In February 2022, NCATS’ CTSA Program joined an effort coordinated by the NIH Office of The Director to support retention of diverse researchers through Administrative Supplements to Recognize Excellence in Diversity, Equity, Inclusion, and Accessibility Mentorship [20]. This award recognizes the crucial role mentors play in the development of future leaders in scientific research. It aims to provide support to scientists with existing NIH awards who are outstanding mentors and leaders, demonstrate compelling commitments to mentoring (especially to individuals from underrepresented groups), and contribute to enhancing DEIA in the biomedical sciences.

### Early NCATS Considerations for DEIA in Translational Science to Address Health Disparities and Inequities

Prioritization of DEIA in translational science focuses on identifying challenges and opportunities to integrate DEIA into research across the translational spectrum. These challenges might be specific to a particular phase of the translational spectrum – such as basic research, clinical research, or population health research – or a more systemic issue that cuts across all research activities. Here, we share information about an internal NCATS working group that has developed an action plan for integrating DEIA across NCATS and its research activities. Then, we highlight four key considerations NCATS has explored for advancing DEIA in translational science, along with examples of NCATS-affiliated activities that reflect these considerations.

### NCATS Health Disparities Working Group (HDWG)

In Summer 2020, the NCATS Director created the Health Disparities Working Group (HDWG) and charged the group with developing an action plan for how NCATS could leverage its activities and national leadership position in translational science toward eliminating racial/ethnic health disparities and advancing DEIA. The HDWG is a coalition of willing and committed staff from all NCATS offices and divisions, crossing all scientific and administrative roles, ranks, races/ethnicities, and genders.

To inform development of the action plan, the HDWG gathered information through an all-staff survey (*n* = 149, 25% of the NCATS workforce) and confidential follow-up interviews (*n* = 27). These methodologies provided staff with an opportunity to share information about NCATS activities that could be leveraged toward a strengthened commitment to investing in DEIA and to identify opportunities for NCATS to break cycles of institutional practices that contribute to the pervasiveness of racism and health inequity. Completed in Spring 2022, the summary findings provided the foundation for developing the HDWG’s action plan. The action plan provides considerations for NCATS leadership and employees to incorporate DEIA into NCATS’ practices to address health inequities and health disparities. These considerations are categorized into three focus areas: (1) fostering an organizational culture that increases awareness and continued learning of DEIA; (2) expanding the NCATS scientific portfolio to prioritize research activities that advance the science on minority health, health disparities, and health equity; and (3) addressing practices to support a diverse workforce at NCATS and the broader translational science community. Implementation of these practices is currently in discussion by IDEA Council and NCATS leadership and will be continuously evaluated by Center staff to assess short-term outcomes and long-term impact. In terms of prioritizing research activities, next we share key strategies identified in NCATS-affiliated initiatives.

### Key Strategies for Advancing DEIA via Translational Science

Translational science has the power to introduce generalizable solutions to systemic problems in the translational pipeline to dramatically advance research toward treatments, interventions, and cures to benefit the public. To advance DEIA in translational science, we can consider generalizable solutions that cut across research initiatives, diseases, and conditions. Here, we highlight four areas often mentioned when discussing health disparities and health inequities, and how they may be applicable for advancing DEIA in translational science, recognizing that these are a sample of approaches that may have relevance in the broader translational science community. Within each of the considerations highlighted here, we share examples of NCATS activities within the Center and through partnership with extramural partners that reflect the consideration.

#### Strategy 1: Encouraging innovation for robust representation of diverse populations in biomedical research

Poor representation of diverse populations in research studies stymies the ability to explore research questions around minority health and health disparities, hinders equitable access to scientific advances, and prevents full understanding of factors contributing to disease or health. Therefore, robust representation of diverse populations in research is a translational science priority for advancing DEIA. There are various challenges to including diverse populations in research across the translational spectrum, and translational science can identify these variations and overcome them through tailored strategies.

One example of NCATS investments around inclusion of diverse populations in the drug discovery and development space is a focus on achieving equitable benefits from gene-targeted therapies for rare diseases. In June 2021, NCATS co-hosted a roundtable meeting series on gene-targeted therapies in which equitable access to therapies was a central theme. Representatives from the NIH, FDA, academia, patient advocacy groups, payers, and industry drew attention to current impediments to access gene-targeted therapies. One challenge that contributes to health disparities is the reliance on individual patients, family/friends, patient organizations, and clinical research champions to provide personal resources and capital to support research development.

The roundtable included a presentation of a framework for enhancing equity in gene therapy, including the following four principles: (1) create innovative approaches to provide equitable access; (2) develop intentional engagement strategies for communities who lack resources to access gene-targeted therapies; (3) democratize access to high-quality education materials; and (4) build a trustworthy field of science [21]. Discussions addressed the need for strategies to support equity to consider screening to confirmatory diagnostic testing to treatments and ancillary care. Collectively, presenters described a vision where diverse patients could be equitably connected to appropriate gene-targeted therapies. Strategies discussed in this roundtable have been applied to NCATS activities. For example, NCATS’ Division of Rare Diseases Research and Innovation has partnered with community stakeholders to develop educational multimedia on gene therapy and genome editing for patient audiences [22].

Another collaborative initiative within NCATS’ Clinical and Translational Science Awards (CTSA) Program is the Trial Innovation Network (TIN) [23]. The TIN aims to introduce innovations that resolve critical roadblocks in clinical trials, toward enhancing efficiencies and optimizing research quality and impact. Engagement of diverse populations in research is included as a central aim. The TIN online Toolbox provides a range of resources for community engagement, recruitment, and retention activities for clinical trials. Strongly represented among these resources are items that inform or facilitate efforts toward diverse representation in clinical resource.

#### Strategy 2: Leveraging big data and informatics to advance DEIA in translational research

In this era of big data, there are many opportunities to identify, address, implement, and evaluate the effectiveness of solutions that integrate DEIA into research efforts across the translational spectrum. Inconsistencies and limitations in race/ethnicity data reporting and collection are translational science challenges hindering research on minority health and health disparities and therefore negatively impacting the entire translational spectrum. For example, imprecise and broad categorizations of race lead to information loss, preventing population-level discoveries and clinical decision-making and treatment efforts [24]. Further, application of multiple or different race/ethnicity definitions affects data analytics and produces conflicting conclusions related to disparities [25]. NCATS hosted a workshop regarding the use of Machine Intelligence applications with speakers from various fields including Health Informatics, Bioethics, Public Health, and Engineering to discuss limitations and considerations of MI in the areas of trustworthiness, explainability, usability, and transparency [26].

Limitations also occur earlier in the translation process where preclinical models do not span the broad racial/ethnic groups that are observed in human populations. For example, cancer cell lines used for functional variant modeling knowledge are mostly derived from non-Latino white individuals, and to address cancer health disparities, different modeling techniques may need to be developed [27]. Thus, there is a need to develop considerations in translational science that can be leveraged to address these limitations, including (a) thorough and thoughtful approaches to data collection; (b) reporting that considers quality standards around DEIA; and (c) reporting that enables harmonization with broadly accepted standards. These approaches further benefit when considered through a multi-disciplinary and multi-stakeholder lens. Tackling these challenges will ensure the resources invested in data collection, storage, and integration are maximally leveraged for data analyses with a focus on advancing research on minority health and health disparities.

One example of NCATS investment in informatics and big data is the National COVID Cohort Collaborative (N3C). The N3C is a partnership of NCATS with the CTSA-funded institutions, NCATS-supported National Center for Data to Health, and the National Institute for General Medical Sciences-supported Institutional Development Award Networks for Clinical and Translational Research, (IDeA-CTR). These partners worked together to obtain existing clinical data from COVID-tested patients from over 70 clinical sites across the United States, including research institutions, federally qualified health centers, and community health centers.

As of May 2022, the N3C provides a national research resource for COVID-19 clinical data derived from over 5 million COVID patients, and over 70 data-contributing organizations on symptoms, medical conditions, medical care received, and more [28]. The N3C enclave includes data on patient demographics and access to relevant public datasets such as indices of social determinants of health. The N3C platform enables critical insights into the relative burden of COVID-19 infections, hospitalizations, mortality by race/ethnicity and gender, and other social determinants of health factors. A recent analysis of N3C reported race and ethnicity biases and how these affect the quality of the harmonized data within N3C [29]. Notably, the analysis emphasizes the importance of making transparent the process of data harmonization to enable informed decisions on generating race and ethnicity phenotypes. We note that this transparency is built into the design of N3C and is accessible to users of the system. Collectively, access to data within N3C offers the opportunity to explore effective approaches to treatment and care, which when combined with disparities data offers avenues toward eliminating disparities in COVID – including long-COVID – outcomes [30].

The N3C’s broad outreach allows for data collection representing diverse demographic and geographic groups. NCATS sought a Consultation with Tribal Nation leaders in early 2022 to solicit input on whether and how to appropriately make American Indian/Alaska Native data available to Tribal researchers and to the broader scientific research community [31]. As of September 2022, this data is now accessible for COVID research.

#### Strategy 3: Prioritizing research for the health needs of underserved groups

A critical translational science challenge is addressing the health needs of underserved populations in the United States, which has been historically in need of support as reflected in funding and research [32]. A central tenet of translational science is to prioritize research addressing unmet health needs [33]. Efforts to rectify historical inattention to minority health and health disparities require identification and correction of deficits in how priorities are set at each stage of the translational spectrum.

NCATS is leading or collaborating on efforts to advance research with a focus on health challenges disproportionately impacting underserved populations. For example, NCATS plays a role in the NIH Helping to End Addiction Long-term (HEAL) Initiative, which aims to improve prevention and treatment strategies for opioid misuse, pain, addiction, and problems that have disproportionately burdened underserved populations [34]. NCATS is providing a suite of translational science resources and technical expertise to investigators in HEAL-relevant areas. These include collaborations for developing drugs and human cell-based testing platforms; preclinical testing of candidate therapeutics for pain treatment; developing a specialized platform for innovative research exploration; and using tissue chips to model pain reception, addiction, and overdose [35].

Another NCATS partnership is with NIH RADx-UP (Rapid Acceleration of Diagnostics-Underserved Populations) [36]. RADx-UP aims to understand factors associated with disparities in COVID-19 testing, infection, and severity. Overall, RADx-UP supports more than 125 research projects that are collecting data on disparities in COVID-19 testing, infection rates, disease progression, health outcomes, and access to healthcare. These data will be used to develop strategies for reducing COVID-19 disparities such as improving testing and care for underserved populations. RADx-UP has leveraged the resources and infrastructure of NCATS-supported CTSA institutions, including community partnerships and research participant recruitment infrastructure critical to understanding community needs [37].

#### Strategy 4: Addressing the “Last-mile” translation

Last-mile translation involves ensuring that research findings and the translational applications they produce (e.g., treatments, cures, and interventions) reach the public. A DEIA lens recognizes differential barriers to accessing understandable, accurate, and relevant information on translational findings and applications, and responds with tailored solutions. These barriers for last-mile translation can occur at any point of the translational spectrum to impact how research outcomes reach the public. For example, a DEIA lens would consider only including participants who are not underserved in clinical trials to be a barrier to applicability of approved medications to underserved populations who the disease may disproportionately impact. Another barrier to last-mile translation is access to resources in multiple languages.

NCATS is investing in eliminating disparities related to last-mile translation through increasing equitable access to the Genetic and Rare Diseases (GARD) Information Center, funded by NCATS and the National Human Genome Research Institute. Viewed by a global audience of over 16 million individuals, of whom nearly 12% are Spanish-language speakers, the GARD website aims to provide users (e.g., patients and caregivers) with access to current, reliable, and accessible health information about rare and/or genetic diseases in both English and Spanish. GARD also offers multilingual support to users that request information about rare diseases to translate resources into plain language and/or Spanish.

Systematic reviews for Spanish-language consumer health information technology interventions point to additional strategies for effectively disseminating research findings to diverse audiences [38]. Beyond language, content must also be culturally tailored, and website components (e.g., user interfaces and functionality) should be evaluated for the engagement of diverse populations. This research evidence underlies work currently underway to improve the GARD website. It leverages user experience research to gain knowledge of how the website can be more responsive to the sociocultural and linguistic lenses through which target audiences interpret the included health information.

## Conclusion

NCATS is committed to applying the power of translational science to bring more treatments to *all* people more quickly and to bringing a lens of DEIA into our activities. Along with the NIH, NCATS is examining its current activities, and considering additional efforts and processes to address long-recognized challenges caused by structural racism and advance DEIA across NCATS activities. The examples offered here demonstrate how translational science can be leveraged to advance DEIA in research and ultimately contribute to addressing health disparities and inequities. Internal NCATS considerations will include long-term sustainability and accountability through rigorous evaluation of goals and benchmarks to advance DEIA and ensure that scientific and operational aspects of translational science benefit all people equitably.
